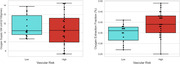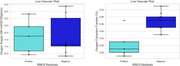# Microvascular physiology differs in older adults with varying vascular risk and white matter lesion burden

**DOI:** 10.1002/alz.093483

**Published:** 2025-01-09

**Authors:** Gabriele M Gassner, Nikou L Damestani, Shrikanth M Yadav, Natalie S Wheeler, John R Jacoby, Sarah F Mellen, Katherine N Maina, David H Salat, Meher R Juttukonda

**Affiliations:** ^1^ Kiel University Faculty of Medicine, Kiel Germany; ^2^ Harvard Medical School, Boston, MA USA; ^3^ Massachusetts General Hospital, Charlestown, MA USA; ^4^ Neuroimaging Research for Veterans Center, VA Boston Healthcare System, Jamaica Plain, MA USA

## Abstract

**Background:**

White matter lesions (WMLs) are common with aging and are prevalent in AD, but the underlying physiology as well as associations with conventional vascular risk factors are not yet fully understood. In this study, we investigated the relationship between vascular risk factors and microvascular physiology (i.e., oxygen supply and oxygen extraction fraction), and their association with WML burden in older adults.

**Method:**

Study design: Typically‐aging older adults between 60–80 years (n=42) were enrolled and categorized as ‘high’ (VRF+; n=27) or ‘low’ vascular risk (VRF−; n=15) based on medication use, clinical diagnosis, and disease‐specific markers of four modifiable conditions: hypertension, diabetes, hyperlipidemia, and overweight. Participants were further subdivided into ‘high’ or ‘low’ WML burden based on positive or negative residuals from a regression of WML burden against age.

Hemodynamic imaging: Magnetic resonance imaging (MRI) data was acquired using pseudo‐continuous arterial spin labeling (ASL) for cerebral blood flow (CBF) and T_2_‐relaxation‐under‐spin‐tagging (TRUST) MRI for oxygen extraction fraction (OEF). Cerebral blood blow (CBF) was computed using a two‐compartment model and with accounting for arterial transit time. Cerebral oxygen supply was computed as the product of CBF, arterial oxygen saturation, and the oxygen transport capacity of blood. Venous oxygenation was derived from TRUST, and OEF was computed as the relative ratio between arterial oxygen saturation (from pulse oximetry) and venous oxygenation.

**Result:**

Participants did not differ in oxygen supply, but OEF was significantly higher in VRF+ (39.22%) versus VRF− (35.2%) individuals (p = 0.02; Figure 1). Within the VRF+ group, no differences were observed in oxygen supply or OEF were found when comparing high versus low lesion burden individuals. Within the VRF− group, no differences in oxygen supply were observed, but OEF was significantly lower in the high lesion burden (30.4%) compared to the low lesion burden (37.6%) subgroup (p = 0.01; Figure 2).

**Conclusion:**

Our findings suggest that lower OEF may represent a marker of impaired physiology associated with WML burden in the absence of conventional vascular risk factors. Such impairment could arise from disturbed capillary transit patterns, and future work will investigate these mechanisms.